# Using smart‐messaging to enhance mindfulness‐based cognitive therapy for cancer patients: A mixed methods proof of concept evaluation

**DOI:** 10.1002/pon.5256

**Published:** 2019-11-25

**Authors:** Chloe Wells, Sam Malins, Simon Clarke, Iwona Skorodzien, Sanchia Biswas, Tim Sweeney, Nima Moghaddam, Jo Levene

**Affiliations:** ^1^ Department of Psychology Nottingham Trent University Nottingham UK; ^2^ Institute of Mental Health University of Nottingham Nottingham UK; ^3^ Specialist Services Nottinghamshire Healthcare NHS Foundation Trust Nottinghamshire UK; ^4^ School of Psychology University of Leicester Leicester UK; ^5^ School of Psychology University of Lincoln Lincoln UK

**Keywords:** cancer, cognitive therapy, dropout, mindfulness, oncology, telehealth

## Abstract

**Objective:**

Depression and anxiety lead to reduced treatment adherence, poorer quality of life, and increased care costs amongst cancer patients. Mindfulness‐based cognitive therapy (MBCT) is an effective treatment, but dropout reduces potential benefits. Smart‐message reminders can prevent dropout and improve effectiveness. However, smart‐messaging is untested for MBCT in cancer. This study evaluates smart‐messaging to reduce dropout and improve effectiveness in MBCT for cancer patients with depression or anxiety.

**Methods:**

Fifty‐one cancer patients attending MBCT in a psycho‐oncology service were offered a smart‐messaging intervention, which reminded them of prescribed between‐session activities. Thirty patients accepted smart‐messaging and 21 did not. Assessments of depression and anxiety were taken at baseline, session‐by‐session, and one‐month follow‐up. Logistic regression and multilevel modelling compared the groups on treatment completion and clinical effectiveness. Fifteen post‐treatment patient interviews explored smart‐messaging use.

**Results:**

The odds of programme completion were eight times greater for patients using smart‐messaging compared with non‐users, controlling for age, gender, baseline depression, and baseline anxiety (OR = 7.79, 95% CI 1.75 to 34.58, *p* = .007). Smart‐messaging users also reported greater improvement in depression over the programme (*B* = ‐2.33*, SEB* = .78, *p* = .004) when controlling for baseline severity, change over time, age, and number of sessions attended. There was no difference between groups in anxiety improvement (*B* = ‐1.46*, SEB* = .86, *p* = .097). In interviews, smart‐messaging was described as a motivating reminder and source of personal connection.

**Conclusions:**

Smart‐messaging may be an easily integrated telehealth intervention to improve MBCT for cancer patients.

## 1 BACKGROUND

Cancer patients are at three times the risk of depression and anxiety during and after their treatment.^1^, ^2^ Depression and anxiety lead to reduced effectiveness of and adherence to cancer therapies, poorer quality of life and significantly increased cancer care costs.^3^, ^4^

Mindfulness‐based cognitive therapy (MBCT) is a well‐evidenced treatment for depression and anxiety among cancer patients.^5^, ^6^, ^7^, ^8^, ^9^, ^10^, ^11^, ^12^, ^13^ This group‐based programme teaches participants to purposefully attend to their experiences in the moment, just as they are, without judgement.^5^, ^14^ This philosophy can be helpful for cancer patients dealing with difficulties and uncertainties around their current or future health. However, MBCT requires substantial prescribed home practice activities between sessions and non‐adherence can lead to poorer outcomes.^15^ Furthermore, dropout rates in recent trials of mindfulness‐based group interventions in cancer have ranged from 21% to 35%,^16^, ^17^, ^18^ which is higher than for group psychological therapies in general.^19^, ^20^, ^21^ The problem may be particularly acute amongst cancer patients because of physical limitations and ongoing treatment. However, an eHealth enhancement could be used to improve the situation.

Current evidence suggests that brief, easily‐implemented reminders, such as targeted text‐messages, can reduce non‐attendance and improve health behaviour‐change.^22^, ^23^, ^24^ “Smart‐messaging” interventions like this have been shown to improve physical and mental health among cancer patients.^25^ Yet, smart‐messaging has not been used for MBCT in cancer, despite evidence that smart‐messaging is one of the easiest and most cost‐effective telehealth interventions to integrate within routine care.^22^, ^26^, ^27^

This study aimed to evaluate the use of a smart‐messaging intervention to reduce dropout and improve effectiveness of MBCT for cancer patients with depression and anxiety. The study also aimed to evaluate the effectiveness of MBCT for cancer patients in a routine care setting.

## 2 METHOD

### 2.1 Design

A mixed‐methods proof of concept evaluation was used to offer initial assessment of smart‐messaging's effectiveness in MBCT for cancer. The study also explored potential mechanisms of change. The smart‐messaging reminder intervention + MBCT was compared with MBCT alone. Smart‐messaging was added to six consecutive groups of MBCT run between November 2015 and July 2018 for cancer and palliative care patients who were referred to a UK psycho‐oncology service. Each patient was offered the choice of receiving smart‐messaging reminders during MBCT or not. Users and non‐users were compared on MBCT completion rate and clinical outcomes, controlling for baseline severity and key demographic characteristics.

### 2.2 Participants

Participants were receiving cancer treatment from an NHS hospital or palliative care from a hospice and had been referred to the embedded psycho‐oncology service. All 51 patients who attended one or more sessions of MBCT were included in the study*. Thirty patients chose to receive smart messaging during the programme and 21 patients did not. Patients were told that they did not need to give a reason for their choice and were not asked why they declined to avoid clinician coercion. Fifteen participants took part in post‐treatment interview; those who had not used smart‐messaging were then asked about reasons for declining.

### 2.3 Procedure

All patients were referred to psycho‐oncology by healthcare professionals from their cancer care team, including oncologists, surgeons, radiologists, cancer nurse specialists, and allied healthcare professionals.

Patients were assessed by a clinical psychologist and accepted into MBCT if at least mild‐to‐moderate clinical anxiety and/or depression symptoms were identified using standardized assessments. Patients were included if their overall health status suggested that they would be able to attend and participate for the full duration of the programme. If patients were too unwell for the programme they were offered one‐to‐one psychological support. At initial assessment the commitments required during the programme were described. This included the commitment to carry out home practice mindfulness exercises for 40 minutes six days‐a‐week between sessions and attend all sessions (two planned omissions were accepted, as many patients still required several other treatment appointments). If patients were unable to meet these commitments, they were not included in an MBCT programme, but offered a different psychological therapy. In total 283 patients were referred to the psycho‐oncology service in the study period and 76 (27%) patients were assessed as suitable for the MBCT programme. The 25 who did not attend any sessions of MBCT withdrew primarily for health‐related limitations (e.g. feeling too fatigued, 10/52) or for practical reasons (e.g. unable to commit to programme dates or times 6/25). The remainder either experienced spontaneous remission prior to the group starting and opted out (4/25) or gave no reason (5/25).

At initial assessment, the smart‐messaging reminder intervention was explained to all patients. Written informed consent was then sought from patients who agreed to use smart‐messaging, ensuring patients understood that:

1. The system was automated (without a real person to respond in case of emergency)
2. Anonymized messaging data would be used for evaluation
3. They could stop the messages at any time

The care offered to those who opted‐out of receiving smart‐messages was not changed in any other way.

Standardized, self‐reported pre‐group outcome assessments were completed by patients between one and four weeks prior to the start of MBCT. Assessments were then completed prior to each MBCT session, including one‐month follow‐up. Session attendance was recorded on a patient register maintained by the programme leaders.

Unique identifiers were used on the smart‐messaging system instead of names to maintain anonymity. The system stored data on patients' use of smart‐messaging, which was linked to the appropriate patients using a key to the unique identifiers stored securely by the psycho‐oncology service. Demographic and clinical details were extracted from patients' clinical records and compiled on an anonymized database.

All patients were invited to take part in a 15‐minute telephone interview about their experiences of MBCT including questions about their experience of smart‐messaging (e.g. “How did you find the text‐messages you received as part of the programme?”). Those who declined to use smart‐messaging were asked about their reasons for opting‐out. Interviewees gave written informed consent for the interview to be recorded, transcribed verbatim and the contents anonymously reported. Fifteen‐minute telephone interviews were completed in the two weeks after the one‐month follow‐up session by research assistants and students who were not involved in leading the MBCT programme. Interviewers were trained in semi‐structured interview technique and were familiar with an interview guide used to focus the interview on key areas of interest.

### 2.4 Outcome measures

Programme completion was the primary study outcome. Programme completion has previously been defined as attending four or more MBCT sessions, because this has been observed to be the adequate treatment dose for clinical effects.^28^ Programme completers were defined in this way for analysis. Session attendance was defined attending any part of an MBCT session, even if late. Attendance was recorded by MBCT therapists.

Symptom improvements were secondary outcomes. The nine‐item Patient Health Questionnaire (PHQ‐9) measures symptoms of depression based on diagnostic criteria for major depression. The PHQ‐9 has excellent test‐retest reliability (α = .84) and good convergent validity with general mental health assessment (*r* = .73).^29^

The seven‐item Generalized Anxiety Disorder scale (GAD‐7) is a measure of generalized anxiety symptoms based on diagnostic criteria. It has excellent test‐retest reliability (ICC = .83) and good convergent validity with other anxiety measures (*r*s = .72 to.74).^30^

### 2.5 Interventions

#### 2.5.1 MBCT

During MBCT participants are trained to use mindfulness skills to address problems related to ruminative thinking patterns known to maintain anxiety and depression.^5^ The programme particularly focuses on the use of non‐judgemental, present moment awareness to make purposeful choices about self‐management of physical and emotional health.^5^, ^31^ Each session involves reviewing activities carried out between sessions; introducing new mindfulness practices; exploring in‐session experiences, and psychoeducation based on cognitive therapy^32^ around the role of mindfulness in everyday life. The programme runs for eight weekly sessions with an additional one‐month follow‐up session. An adapted version of the MBCT programme was used to address the needs of cancer patients.^31^ The cancer‐adapted version of MBCT closely follows the original programme, but makes explicit reference to common problems associated with cancer and its treatment.

#### 2.5.2 Smart‐messaging

The “Florence” eHealth system (http://www.simple.uk.net) was used to provide MBCT attendees with text‐messages to support their attendance and engagement. Florence would send a text‐message the day after the MBCT session reminding participants of the home practice for the week:

Hi, it's Jo & Sam†. This is a reminder of the week's home practice: Body scan, routine activity, the pause, eating mindfully. Text Y for more.

Patients receiving the text could then request up to four further texts by replying with “Y” which offered more detail on the initial text:

Hi, it's Jo & Sam. The body scan is about allowing things to be as they are without trying to changethem. Text Y for more.

Two days later the Florence system sent a reminder of the theme for the previous session:

Hi, it's Jo & Sam. This is a reminder of the theme from week 1 – we looked at the impact of being on automatic pilot. Text Y for more.

Again, at patients' choice they could receive no further messages or request up to four further reminders which added more detail:

Hi, it's Jo & Sam. On automatic pilot we are more likely to react out of habit which often means we are stressed more easily. Text Y for more.

Lastly, patients were sent a reminder about the following session on the day before the group was due to meet:

Hi, it's Jo & Sam. This is a reminder that we are meeting tomorrow morning, so today is the last opportunity to practice! Text Y for more.

Patients received a minimum of three text‐messages per week but could request an additional nine if they wished.

### 2.6 Therapists

The MBCT therapists were trained using a national programme which included attending an MBCT programme, leading assessed MBCT sessions, leading an initial staff MBCT programme, and monthly supervision with an experienced MBCT therapist.

### 2.7 Method of analysis

The overall clinical effectiveness of MBCT was evaluated using paired t‐tests comparing baseline depression (PHQ‐9) and anxiety (GAD‐7) with one‐month follow‐up. Cohen's *d* 33 was calculated for a standardized effect size.

Patient characteristics were compared between smart‐messaging users and non‐users. Categorical variables were compared using chi square and continuous variables using Mann‐Whitney U.

The comparative odds ratio for MBCT completion was calculated using a logistic regression with MBCT completion as a dichotomous outcome whilst controlling for age, gender, baseline depression and baseline anxiety.

To account for the repeated measurement design, multilevel modelling was used to assess differences between smart‐message users and non‐users in anxiety and depressive symptoms over time. Multilevel modelling allows data to be nested hierarchically. In this case, repeated measurements of depression or anxiety (level 1: *time* hereafter) were nested within the individual patients who gave those measurements (level 2). Multilevel modelling therefore accounts for covariance of depressive symptoms occurring *within* participants and *between* participants compared with more typical regression methods.^34^ Covariance within participants accounted for 79% of total covariance. Established model‐building guidelines were used to develop the multilevel analysis.^35^ The hierarchical structure of data was examined in unconditional models, then model fit was explored using ‐2 log likelihood. Random intercepts and slopes were included for *time* and a diagonal covariance matrix was used. Parameter estimation was made using maximum likelihood. When depression (PHQ‐9) was the outcome, the fully adjusted model included the following fixed‐effect covariates at the patient level: *time* (adjusting for ordered change over time); *baseline PHQ‐9 score* (adjusting for baseline depression severity); *number of sessions attended* (adjusting for MBCT dosage); and *age* (controlling for demographic differences in engagement with smart‐messaging). A parallel model was applied to examine anxiety (GAD‐7) as the outcome variable: The same model development methods and covariates were repeated but assessing whether use of smart‐messaging predicted anxiety.

As data were collected from routine care, sample size could not be manipulated. However, the sample size exceeded the minimum recommended for both multilevel modelling and logistic regression (minimum n of 50 at level 2 in multilevel modelling and minimum n of 10 per parameter in regression analyses^36^, ^37^;). Missing data were not imputed, only observed values were used in analysis.

Semi‐structured interviews were analysed by first extracting discussion of smart‐messaging from interview transcripts then using thematic analysis.^38^ This included making initial notations, followed by grouping notations into themes and then structuring these themes as succinctly, coherently and meaningfully as possible. An independent review of the analysis was carried out by one author who had not been involved in data collection or initial analysis (SM), to enhance the quality and dependability of findings.

Ethical approval was not required, as the study used anonymized data collected as part of routine care. The study was registered with the host NHS Trust.

## 3 RESULTS

### 3.1 Patient characteristics

The majority of patients were women (84%), with breast cancer being the most common cancer site (49%). The patients who chose to use smart messaging were significantly younger than non‐users (mean age = 54 versus 59; Z = 2.12, *p* = .034). Smart‐messaging users also attended more sessions (mean = 6 versus 4; Z = 3.55, *p*<.001). These factors were entered as covariates in models comparing the clinical effectiveness of the two groups. Smart‐messaging users did not differ from non‐users on any other characteristics (Table S1).

### 3.2 MBCT completion rate

The odds of MBCT completion (attending four or more sessions) were eight times higher for smart‐messaging users than non‐users, when controlling for age, gender, baseline depression and baseline anxiety (OR = 7.79, 95% CI 1.75 to 34.58, *p* = .007; Table 1). Twenty‐six smart‐messaging users (87%) completed MBCT compared with eight non‐users (38%).

**Table 1:** Smart‐messaging use as a predictor of MBCT completion

Predictor variable	*B*	*SE*	Wald	*p*	Adjusted OR	95% CI	
Age	‐0.03	0.04	0.67	.437	0.97	0.91	1.04
Gender	1.04	1.13	0.85	.413	0.97	0.90	1.04
Baseline depression (PHQ‐9)	‐0.08	0.09	0.69	.357	2.82	0.31	25.65
Baseline anxiety (GAD‐7)	‐0.06	0.10	0.43	.407	0.93	0.78	1.11
*Smart‐messaging use*	*2.05*	*0.76*	*7.28*	*.007*	*7.79*	*1.75*	*34.58*

### 3.3 Overall effectiveness

**Figure 1:**
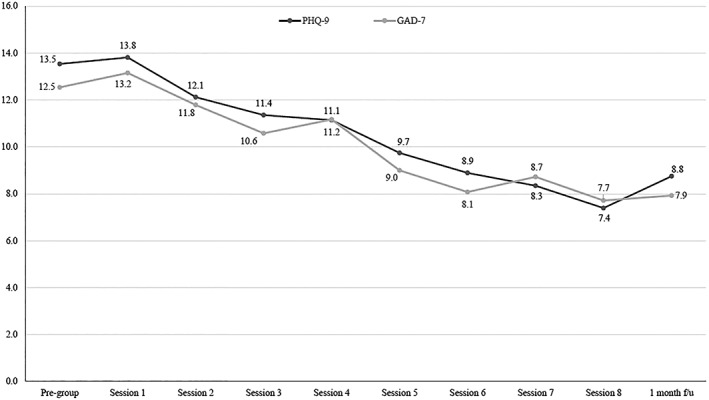
Depression and anxiety scores over time

Paired t‐tests indicated a significant reduction in symptoms of depression (PHQ‐9: *M* = ‐4.7, *SD* = 4.6; *t*(27) = 5.59, *p* < .001) and anxiety (GAD‐7: *M* = ‐4.6, *SD* = 5.7; *t*(26) = .88, *p* = .001) from baseline to one‐month post‐treatment. This suggests a large pre‐post effect size for depression (*d* = .8) and anxiety (*d* = .9). There were also trends for stepwise improvements in symptoms as more MBCT sessions were attended (Figure 1).

Figure 1. Depression (PHQ‐9) and anxiety (GAD‐7) scores over time

### 3.4 Differential effectiveness

Smart‐messaging users showed significantly reductions in symptoms of depression over the MBCT programme compared with non‐users (Table 2). Depression scores (PHQ‐9) reduced by 2.3 points (95% CI: 0.76 – 3.89) more amongst smart‐messaging users, when controlling for baseline severity, age, number of sessions attended, and covariance over time. Smart‐messaging users did not differ significantly from non‐users on changes in anxiety, but there was a trend for greater improvement (*B* = ‐1.46*, SE B* = .86, *p* = .097).

**Table 2:** Smart‐messaging use as a predictor of depression change over time

Fixed effects						Random effects				
				95% CI						
Parameter	*B*	*SE*	*p*	Lower Bound	Upper Bound	Parameter	Variance	*SE*	*z*	*p*
Intercept	5.06	2.65	0.062	‐0.27	10.38	Residual variance	6.64	0.60	11.08	0.000
Assessment time point	‐0.67	0.10	0.000	‐0.87	‐0.47	Intercept variance	1.62	1.09	1.49	0.137
Baseline depression (PHQ‐9)	0.87	0.06	0.000	0.74	0.99	Slope variance	0.27	0.09	2.99	0.003
Number of sessions attended	0.23	0.17	0.173	‐0.10	0.56					
Age	‐0.04	0.03	0.265	‐0.10	0.03					
*Smart‐messaging use*	*‐2.33*	*0.78*	*0.004*	*‐3.89*	*‐0.76*					

### 3.5 Smart‐messaging engagement

Smart‐messaging users requested further messages 14 times on average during MBCT (*SD* = 16; range = 0–50, a total of 63 messages could have been requested).

### 3.6 Patient interviews (n=15)

Two patients were interviewed who had opted not to use smart‐messaging. Both explained that confidence in the use of mobile phones had prevented them from using it:

I don't really read messages on my phone or anything like that [] sometimes I don't really understand it fully so, and I don't like to ask 'cos I feel daft, if you know what I mean, it's like you should know everything apparently according to my kids anyway. I should be in the know about everything about computers and all that, and I'm not so.

(Group 5; patient D)

From interviews with 13 patients who used smart‐messaging, one theme was represented in all interviews: smart‐messaging as a *prompt and reminder*:

Yes, I thought [smart‐messaging] was a good idea, they reminded you [] they prompted you to think: “Yes, I'll do that”. (Group 2, patient A)

In some cases the reminding effect was also motivating:

I thought they were good, erm it sort of motivated you and was a gentle reminder really, yeah. So I thought that that was a good idea. (Group 5, patient F)

In other cases, the reminding effect was seen to draw participants back to mindfulness practice:

[Smart‐messages] were good reminders. If you found yourself getting lost they were good reminders to put you back on track. Because at the beginning as I say it feels more like homework, you don't feel like you're getting anything from it at the beginning. So it's about putting it into life and doing it and then you get out what you've put into it [] but you don't at first you just think it's homework. (Group 1, patient H)

These quotes suggest the effect of smart‐messaging may be to increase motivation and help MBCT attendees stay “on track” by incorporating mindfulness exercises into their daily lives, particularly during the early phases of the programme when they may not have experienced benefits from home practice. However, a secondary theme of *personal connection* indicated that for some patients (4/13) smart messaging had more than a reminding effect:

When you got it you felt “oh somebody is thinking about me.” Even though it was just a text‐message, if that makes sense. (Group 1, patient I)

Interestingly, the personal connection was still reported even in full acknowledgement that messages were not personally sent:

You felt like, I mean I know they were automated, but in a silly way it felt like somebody was motivating you. I enjoyed them actually. (Group 1, patient D)

These quotes imply that some patients felt a connection to a person, in spite of knowing that the messages were automated. This sense of personal connection again appeared to play a role in motivating patients to do mindfulness home practice.

One patient reported irritation from smart‐messaging:

I kept getting [text‐messages] I think it needs to be sorted out a bit better. (Group 5, patient A)

## 4 DISCUSSION

This paper presents a proof of concept evaluation for a smart‐messaging enhancement to MBCT, pragmatically assessed in routine care. Results suggest that those using smart‐messaging have significantly better completion rates and improvements in depressive symptoms. This study can inform effect and sample size estimates for a future RCT to evaluate clinical efficacy. Among patients who tried the intervention, there are indications of feasibility and acceptability. However, 41% chose not to receive the intervention. Interview data offer an exploratory view of possible mechanisms for the effect of smart‐messaging. Patients predominantly reported that smart‐messages acted as a prompt and reminder, which motivated them to carry out home practice at times when they may not have experienced benefits from this discipline. However, some also reported a more personal connection through the messages they received which further motivated home practice. It is plausible that those who committed to more home practice were less likely to dropout. Overall, this study suggests that integrating smart‐messages about MBCT content is a promising, cheap method worthy of further investigation in improving clinical effectiveness and efficiency.

### 4.1 Relationship to existing research

This study supports existing evidence that relatively brief, low‐intensity behavioural reminders may lead to significant changes in health‐related behaviour amongst cancer patients.^25^ Yet, this appears to be the first application of smart‐messaging technology to MBCT for cancer patients. The current study adds that MBCT benefits may be enhanced by integrating messaging‐based reminders of MBCT teaching and practices. This study's pragmatic, service‐level, choice‐based design offers findings that are reflective of clinical practice and may therefore be more generalizable. Interviews also illuminated possible causal explanations from patients' personal experience.

### 4.2 Study limitations

Uncontrolled group allocation means that the findings reported remain tentative, because selection bias could mean there were unmeasured differences between groups. For example, there was no baseline measure of patient motivation when greater motivation might explain patients choosing to use smart‐messaging, improved completion rates and better outcomes. Nonetheless, several key baseline characteristics were controlled in the analysis.

Despite giving a service‐level impression of the impact smart‐messaging may have in a psycho‐oncology setting, this study can give little specific information for patients from individual cancer sites, stages or treatment types. Therefore, questions remain about the differential impact of smart‐messaging between cancer types.

### 4.3 Future research

Current findings require evaluation within an RCT, including assessment of home practice to evaluate whether smart‐messaging leads to an associated increase in this potential mediator. The smart‐messaging method could be enhanced in future by sending more individualized messages, which can increase the impact.^39^

### 4.4 Clinical implications

Although more focus is given to remote delivery and computerized versions of psychological therapies in psycho‐oncology, this study suggests that existing treatments may be enhanced quickly and cheaply without additional therapist input. Specifically, smart‐messaging is an accessible and easily integrated intervention that may enhance treatment effectiveness and adherence, but can be overlooked as old or peripheral technology. This study suggests smart‐messaging could be a means of enhancing MBCT in psycho‐oncology.

## 5 CONCLUSION

The effectiveness and efficiency of MBCT may be enhanced by integrating smart‐messaging through the programme.

## CONFLICT OF INTEREST STATEMENT

None.

## Supporting information

Table S1.
Patient Characteristics

## Data Availability

The quantitative data that support the findings of this study are available from the corresponding author upon reasonable request. Qualitative data are not publicly available due to privacy and ethical reasons.

## ENDNOTES

*
Two non‐cancer palliative care patients attended the included programmes but are removed from the analysis.

†
Authors SM and JL led all MBCT groups so all texts used their first names as a greeting.
