# Supplementary material for: Using smart‐messaging to enhance mindfulness‐based cognitive therapy for cancer patients: A mixed methods proof of concept evaluation
Source: Psychooncology. 2019 Nov 25;29(1):212–9. doi: 10.1002/pon.5256 (PMC7004102; doi:10.1002/pon.5256)
Supplement: Supplementary file 1 — Table S1. Patient Characteristics [file PON-29-212-s001.docx]

|  | **Smart-messaging received**  **(*n* = 30)** | **No smart-messaging**  **(*n* = 21)** |
| --- | --- | --- |
| *Demographics* |  |  |
| Female (%) | 25 (83) | 18 (86) |
| Age - years (SD) | 54 (8) | 59 (13) |
| *Clinical characteristics* |  |  |
| Baseline depression - PHQ-9 (SD) | 12.9 (5.0) | 15.7 (6.5) |
| Baseline anxiety - GAD-7 (SD) | 12.7 (5.1) | 13.3 (5.5) |
| Number of sessions attended (SD) | 6 (2) | 4 (2) |
| *Cancer site (%)* |  |  |
| Breast | 13 (43) | 12 (57) |
| Gastro-intestinal | 8 (27) | 4 (19) |
| Head and neck | 5 (17) | 1 (5) |
| Haematology | 2 (7) | 1 (5) |
| Urology | 0 (0) | 2 (10) |
| Brain and neuroendocrine | 2 (7) | 1 (5) |

**Table 1. Patient Characteristics**
